# Analysis of the Indirect Costs of Rheumatoid Arthritis in Romania

**DOI:** 10.1155/2019/9343812

**Published:** 2019-06-26

**Authors:** Cătălin Codreanu, Corina Mogoșan, Claudiu Popescu, Marian-Sorin Paveliu

**Affiliations:** ^1^“Dr. Ion Stoia” Clinical Center of Rheumatic Diseases, Bucharest, Romania; ^2^“Carol Davila” University of Medicine and Pharmacy, Bucharest, Romania; ^3^“Titu Maiorescu” University, Faculty of Medicine, Bucharest, Romania

## Abstract

Rheumatoid arthritis (RA) is associated with increased costs generated by resource utilization and loss of work productivity. We have studied 206 RA patients and analyzed indirect costs of RA in Romania (estimated using the human capital approach) in comparison with reported data for other countries. Data were collected using self-reported questionnaires. The average age at inclusion was 55 years, with mean disease duration of 9.4 years; 55 patients had permanent work disability due to RA; 6.35 days of sick leave per patient were reported for the entire year of follow-up; the cost of permanent work disability was 1256€ per patient. From a societal perspective, the average indirect costs for a patient with RA were 1506€, significantly lower than the ones reported by other countries, especially due to the low monetary value of paid work.

## 1. Background

Rheumatoid arthritis (RA) is the most frequent inflammatory joint disease with a prevalence of 0,72% according to data published in 2010. [1]. It has a significant impact on the general health of those affected and also prompts major costs because of substantial productivity loss, with a negative impact at both the individual and the societal level.

Cost studies have become fundamental tools for the optimization of resource allocation by authorities and also for designing effective treatment strategies in RA. Nevertheless, the interpretation of the results can represent a real challenge due to heterogeneity in study methodologies. In most studies, costs are interpreted from a societal perspective, although several analyses have been focused on patients' or payers' perspective. Generally, the data included in these studies have been based on self-report questionnaires, administered every 6 to 12 months [2–7]. A small number of studies have used data from RA registries and administrative sources [8, 9] or medical files. As for the study design, retrospective studies [10], cross-sectional studies [8, 9], and prospective studies [2, 11–13] have been reported. Therefore, published data in the literature reveal the economic impact of RA, based on different sample sizes and representative of different populations. Depending on the cost models used in the analysis, these data are relevant for certain geographic areas and for various medical systems, thus making impossible the extrapolation to other socioeconomic or medical systems. For Eastern European countries, more research is required, which should consider both the resources and the expenses to allow a correct evaluation of the cost of illness for RA.

In the balance of costs attributable to RA, direct and indirect costs can be distinguished.

Direct costs can be classified into direct medical costs generated by the utilization of health care resources, which comprise costs of diagnosis and treatment of the disease, including the services of hospitalization and rehabilitation, and direct nonmedical costs, which include transport expenses to and from the doctor's office, as well as other medical services, like informal care [14].

The indirect costs, or productivity costs, are generated by temporary or permanent work disability, as well as all the consequences induced by the loss of work productivity [14]. The evaluation of indirect costs includes three methods [15].

The friction cost method estimates the value of human capital when another person from the unemployment pool replaces the present value of a worker's future earnings until the sick or impaired worker returns or is eventually replaced. This period refers to the time theoretically necessary for the replacement of an ill employee with another person from the general population, which is available for work, but not yet employed. The friction period varies in time and according to the market places.

From the economic perspective, the term capital refers to one of the factors of production employed to produce valuable and usable goods or services. The human is the subject to take charge of all economic behaviors including transaction, consumption (mainly in a market of goods), and production (in a market of inputs or factors of production). So, it can be recognized that human capital means one of the production factors or inputs that can generate additional values by employing it into a production process [15].

The human capital approach estimates productivity costs by taking into account the whole period during which a sick employee is unable to deploy a paid or nonpaid activity, from the first day of absence on sick leave until the last one (the day of resuming work, the day of retirement on illness grounds, or the day of decease) [16]. To promote the standardization of data collection, a list of areas of interest was issued, serving as an indicative matrix approach of the RA economic impact [17] (Table 1).

The willingness to pay method measures the amount that an individual is eager to pay in order to reduce the probability of illness or mortality. There are various methods to determine and estimate an individual's willingness to pay, such as conducting surveys, examining the extra wages for highly risky jobs, and examining the demand for products that leads to greater level of health or safety [15, 18].

In the absence of appropriate treatment, most active cases of RA evolve towards irreversible joint damage [19] which leads to a major degree of disability. RA treatment requires an intensive approach according to the “treat to target” strategy [20]. This involves precocious, aggressive treatment of the active forms of the disease, aiming for disease remission as the treatment objective, meaning a complete control of the symptoms and of the inflammatory process in order to prevent irreversible destructive joint lesions. To achieve this therapeutic objective, the present treatment recommendations propose the initial use of conventional synthetic disease-modifying antirheumatic drugs (csDMARDs; small molecules, obtained by chemical synthesis, usually with a low price), followed by biological DMARDs (complex pharmaceutical preparations, based on biotechnology in cell cultures, at high costs) only for cases where csDMARDs were inefficient (the patient did not attain disease remission) or where they produced significant side effects [21].

In developing countries, including Romania, healthcare system resources are limited and cannot cover the cost of treatment for all RA patients who would benefit from biological therapy. Consequently, Romanian regulatory agencies (Ministry of Health, National Health Insurance House) have implemented administrative criteria which define the access of RA patients to biological therapy within a prescription protocol. These regulations are based only on efficacy criteria; an incremental cost-effectiveness analysis of biologics is still needed. In this context, the study aims to provide an estimation of the indirect costs of RA in a cohort of patients treated with csDMARDs and bDMARDs in Romania. In this article, we wanted to address the issue of indirect costs to be used in cost-effectiveness studies.

To our knowledge, this is the first study of the appraisal of RA costs in Romania, with specific reference to the indirect costs of the disease.

## 2. Methods

### 2.1. Patients and Data

Between December 2013 and December 2014, a cohort of 206 patients was recruited in a prospective study, the procedure being detailed as follows.

All the patients existing in the electronic database of a university tertiary rheumatology center within the stated time period (n = 3951) were screened for the following inclusion criteria: a recorded diagnosis of RA according to each attending rheumatologist, proof of RA-specific treatment with csDMARDs and/or bDMARDs (medical report/letter, reimbursed prescription), age above 18 years, and acceptance to complete a self-reported questionnaire at 6-month intervals and return it by post.

All patients meeting the inclusion criteria (n = 480, 12.1%) were invited to participate in the study and were sent an informed consent letter. Of these, 285 patients (59.4%) responded to the initial invitation letter and 206 (72.3%) of them completed three rounds of self-reported questionnaires (at 0, 6, and 12 months), thus capturing a 12-month period of look-back. An original questionnaire on resource utilization and work productivity was used which included the following items: the number of days of physical inability for the daily activities, the number of people involved in home care and its frequency, the number of hours per day in which other persons are needed, the number of days of sick leave due to RA (responds considered only for employed patients), and information about the year of retirement on sick grounds (in this subcategory only retired patients due to RA were considered).

The components of the indirect costs used the data published by the National Institute of Statistics (INS), as well as reports provided by the Ministry of Labor, Family and Social Protection. The estimation of the indirect cost analysis in this study followed the human capital approach.

The entire responders included 206 completers (for which demographics are presented); the cohort was divided according to therapy in a group treated with csDMARD (monotherapy and combinations) and with biologics, bDMARDs. Five cases without background medication were excluded from the comparative analysis between groups and two other cases that did not answer questions about drugs and could not be assigned to any grouping. Of the 206 cases of the cohort, comparative analysis between groups was performed on 199 cases (because of too small subgroup (no remissive therapy) to be analyzed separately).

### 2.2. Statistics

Continuous variables are reported as mean ± standard deviation (SD), while nominal variables are reported as “absolute frequency (percentage of group).” Differences in continuous variables between subgroups were assessed using bivariate independent-samples t tests, while associations of nominal variables were assessed using *χ*^2^ tests. All the data were analyzed with IBM SPSS version 10 for Windows (SPSS Inc., Chicago). A level of p < 0.05 was chosen for test significance. Romania's currency is the Romanian leu (RON), but the results were expressed in Euros to allow comparisons with published literature data. During the 2014 fiscal year, the annual average exchange rate published by Romania National Bank was 1 € = 4.4446 lei (RON) [22].

## 3. Results and Discussion

### 3.1. General Characteristics

Demographic characteristics of the patient cohort are shown in Table 2. Although the age of patients on bDMARDs was significantly lower, this had no effect upon the status of work activity, as the average age in the cohort was 54.9 ± 12.7 years, indicating participants who are professionally active.

### 3.2. Temporary Work Incapacity

According to National Institute for Statistics, the average gross income in 2014 was 531 €/month [23]; women earned a gross average income of 514 €/month (17.1 €/day), whereas men earned 545 €/month (18.2 €/day) [23, 24].

Sick leaves are issued as a continuous period of time, without considering working week days or nonworking weekends. According to Romanian regulations, the monetary value of the period of work productivity loss by sick leave implies an allowance of 75% of the employee's income. Consequently, the cost of sick leave was 12.8 €/day for women and 13.6 €/day for men. A final multiplication by the length of the self-reported sick leaves during the entire year of follow-up was applied (sick leave induced by RA; absenteeism due to causes other than RA were not considered in calculations). Consequently, the cost of temporary work incapacity due to sick leave used the following formulation: gross value of daily paid work according to gender multiplied by 0.75 multiplied by the number of days of sick leave (Table 3).

### 3.3. Permanent Work Incapacity

In 2014, the Romanian legislation stated that the legal age for retirement was 59 years and 9 months for women and 64 years and 9 months for men [24]. The average pension for sickness incapacity in 2014 was 522 lei (117.5 €) for women and 629 lei for men (141.5 €) [25]. Among the patients who retired before term due to RA in the studied cohort (n = 55), those who reached the legal age of retirement at the time of enrolment or during the observation period were excluded from the costs calculation of the productivity loss by permanent work disability, due to the transfer costs induced by this subgroup. Thus, among the 55 retirements on sickness grounds, 10 patients were excluded from this calculation. For the remaining 45 cases to analyze, the opportunity cost was used, resulting from the difference between average income and average amount of pension, according to sex, multiplied by 12 (months). The resulting figures were 396.5 € for women (514-117.5) and 403.5 € for men (545 – 141.5).

Table 3 illustrates the costs of the work productivity loss for temporary work disability, due to sick leave for the employees, and permanent work disability, due to early retirement, for the patients under the legal age of retirement in Romania.

### 3.4. Unpaid Work

The inability to carry out a work extends to daily activities, requiring the care offered by another person, most often in an informal manner, usually offered by a member of the family. Informal care must be distinguished from permanent specialized care (direct nonmedical cost) and represents the cost of unpaid work of people involved in this support role. Most often, the latter cost is regarded as an indirect cost and we will consider it as indirect in this study [26].

The most used methods to value the shadow price of care which time are the opportunity cost and the replacement cost. The opportunity cost represents the value of the best of the sacrificed chances, which is given up when any choice is made. The care of people suffering from RA by family members (informal care) may entail the cessation of the latter to the relationship with the labour market, permanent or semipermanent (forgone wages). The other way of calculating the value of informal care is using the replacement cost of the service at the market value of that service or by using the market price of a marketed equivalent service. The option of “making” this service by the family instead of buying it looks as if it is more affordable than buying it, being closer to the best alternative option [26, 27]. The permanent informal care is taken over by an informal caregiver (in Romania, these are mostly the family members). There is no specification of household helper in the official Classification of the Occupations in Romania (COR). The occupation of “housemaid” is stipulated in COR, but this profession is subject to a flexible paying system, largely negotiable with the employee. Data from autocompleted questionnaire showed an estimated average household help activity of 4 hours per day. In order to convert this activity into monetary units, we have used the INS report concerning the average gross wage of 3.05 €/hour in 2014. Therefore, a gross amount of 12.2€ is reached (3.05€ x 4 hours), representing the unpaid work of the informal care for usual activities, for each day of work disability.

For the analyzed cohort, the calculations of cost of unpaid work used the following formula: cost of daily household help activity of 4 hours multiplied by the number of days reported. The estimations are illustrated in Table 4, along with the recorded variables which represented the basis of the estimation of the reference points regarding the impact of RA on the indirect costs. Both wage earners and unemployed persons remained in the same category during the 12 months, preserving their jobs and, respectively, staying not employed. Surprisingly, among the patients who were professionally active, the annual temporary work disability was only 6.35 days of sick leave per patient. There were significant differences between groups according to therapy type: 8.5 days of sick leave per year for each active case on csDMARDs versus 1.8 days for cases on bDMARDs (p = 0.05). The difference could be attributed to the higher treatment efficacy of bDMARDs, but given the lack of clinical outcomes, the causality relationship is hard to affirm.

From the societal perspective, the average annual indirect costs generated by a patient with RA, regardless of therapy, are illustrated in Table 5.

The average annual indirect costs (IC) induced by the loss of work productivity, alongside all its consequences following the sickness period, totaled 3968.71€ per patient with RA. Following the human capital approach, the absenteeism generated by the permanent work disability, due to retirement before term, is responsible for 91.62% of total IC. The lost wages generated by the unpaid work rank second with 6.4%. In our country, the temporary work disability by sick leave has a minor contribution to the total IC (1.98%).

The tendency of giving up professional activity after RA diagnosis has been previously reported [28]. According to the presented data, in this cohort the average disease duration towards permanent work disability due to RA was only 5.7 years. During the first postdiagnosis year, 22% of newly diagnosed RA patients were declared as permanently work disabled. An important role in the loss of work productivity is probably still played by the RA patient's social status in our country [29].

The results of published health economics studies in the United States, United Kingdom, Sweden, Spain, Holland, or Italy are not relevant for Romania, mainly because of the differences in their respective healthcare systems, the current medical practices (for instance, apart from many other differences, in our country institutionalization on RA grounds is nonexistent), costs, the financing systems of care, the major differences of gross domestic product (GDP) level [30], and the difference of average income (which is about 10 times lower in Romania than in other states in the European Union [31]). Moreover, the models used for the estimation of the RA costs differ from one study to another. Nevertheless, the literature consisting of health economics studies on RA claims that the IC proportion in the total costs is 50-75% [32–35]. Overall, the cost studies conducted in the United States after 2000 claim that the average TC per RA patient in 2001 is 9519$ [36]. In the same year in Italy, the average annual TC increased as the severity of the disease is higher. The annual average IC [37] follows a significant ascendant curve amongst the four functional classes [37]: class I (normal functionality) generates an IC of 2705€; class II (functionality slightly limited; activities are accompanied by pain) generates an IC of 9566€; class III (alteration functional status allows only self-care) generates IC of 12183€; class IV (severe functional disability; permanent care is necessary) leads to an IC of 17249€. In addition, the TC analyzed without taking into consideration the functional classes was higher for patients with comorbidities [38]. In a nearby country, Hungary, the annual average TC per patient was 4173€ in 2004, from which IC represents 55% [39]. As reported in 2011, the IC in Romania represents only 21.7% of TC (a significantly lower percentage of the illness cost, compared to what had been published before), having an average value of 1506€. The annual average of the total costs for an RA patient [28] is 6950€, within the limits reported by previously published studies. One could state that the IC is dominant in health economics studies developed in Western European and the North-American countries [13], whereas in Romania it has a significantly lower value relative to the TC value. If we refer to the absolute value of the IC, we can notice lower average values in comparison to published studies [40].

*Limitations of the Study*

*The Representativity of the Cohort*. Although in the sanitary unit, from which the study group was extracted, people from all around the country are being admitted, it is expected that their distribution is not representative because there are many other rheumatology centers. On the other hand, there was no possible randomisation of patients, the main criterion of inclusion in the sample being their acceptance to respond to the questionnaire. Currently, a real-time collection of data from all rheumatology centers has begun, which makes it very close to analyze not only representative samples but the entire cohort of patients with RA in Romania. 

*With Regard to the Methodology for Calculating Indirect Costs*. Information on the activities to which the persons offering the information care give up can be investigated more analytically. They will be able to contribute to the establishment of a transfer cost from the state for persons with disabilities due to the RA according to the reality and not generically as it happens today, regardless of the disability disorder. In the near future, it is worth to analyze the extent and the way in which the informal partial care could be compensated in order to delay the retirement application on the case of sickness targeting the request of a permanently paid aid. 

*Regarding the Interference between RA-Induced Disability and Other Comorbidities*. During the analysis of the data we found that in the design we did not pay enough attention to the existing relationship between the evolutionary stage of the disease and the indirect costs, focusing on an indicator that we considered proxy, the type of therapy received. This source of uncertainty can be avoided in future studies.

## 4. Conclusions

The annual IC per RA patient in Romania are lower than the ones reported by other countries from Western Europe and the United States, given the reduced monetary value of paid work in Romania

## Figures and Tables

**Table 1:** The components of the indirect costs associated with RA [17].

Costs of productivity loss	(i) Productivity loss for wage earner patients (disability, sick leave)
	(ii) Opportunity loss (productivity loss of the patient's family members under their care, disabilities that require the adjustment of daily routine)
	(iii) Lost wages

**Table 2:** Baseline socio-demographic characteristics of RA cohort.

	cohort^#^	csDMARDs	bDMARDs	
	(n=206)	(n=129)	(n=70)	p^&^
age (y)	54.9 ± 12.7	56.8 ± 12.3	51.8 ± 11.8	<0.001
women	178 (86.4%)	114 (88.4%)	59 (84.3%)	0.211
men	28 (13.6%)	15 (11.6%)	11 (15.7%)	0.322
urban dwellers	136 (66.0%)	83 (64.3%)	47 (67.1%)	0.125
actively working	60 (29.1%)	38 (29.5%)	21 (30.0%)	0.236
pensioners	143(69.4%)	89 (69.0%)	49 (70.0%)	0.332
unemployed	3 (1.5%)	2 (1.6%)	0 (0.0%)	0.118
disease duration (y)	9.4 ± 8.9	8.2 ± 8.9	11.3 ± 8.3	<0.001
latest treatment duration (y)	2.7 ± 2.6	2.7 ± 2.9	2.7 ± 2.2	0.226

*Notes*: continuous variables are reported as “mean ± SD”; nominal variables are reported as “absolute frequency (percentage of group)”; the unemployed were excluded from the productivity evaluation.

**Table 3:** Work productivity loss by temporary and permanent incapacity.

n=59	days of sick leave (n/year)	sick leave allowance (€/day)	temporary work incapacity cost (€/year)	retired due to RA (n)	average opportunity cost (€/month)	permanent work incapacity cost (€/year)
Women	337	12.8	4313.6	40	396.5	190320
Men	25	13.6	340	5	403.5	24210
*Total*	362	-	*4653.6*	45		*214530*
*Total cost of work productivity cost lost/cohort (€/year)*					*219183.6*	

*Note*: during the 2014 fiscal year, the annual average exchange rate published by Romania National Bank was 1 € = 4.4446 lei [22].

**Table 4:** The cost of unpaid work and indirect cost inducers in RA.

*(1) the cost of unpaid work*		
(i) number of days of inability for daily activities	/cohort	4139 days
(ii) self-reported daily household of help activity		4 hours
(iii) cost of unpaid work (cost of lost wages) (€)	/patient/day	12.2
	/patient/year	253.74
	/cohort	*50495.8*

*(2) characteristics of pensioners group (n=143)*		
loss of work productivity: retired due to RA		55 (38.5%)
mean age of retirement (y)		48 ± 5.3
sickness retirement after RA diagnosis (y)		5.7 ± 6.0
sickness retirement in the first year after RA diagnosis		12 (22%)

**Table 5:** The average annual indirect costs of RA per patient.

cost/patient/year as cohort	€^*∗*^	% of cost category
unpaid work cost	253.74	6.4 % of IC
temporary work disability cost (sick leave)	78.87	1.98% of IC
permanent work disability cost (sick retirement)	3636.1	91.62% of IC
total indirect costs /patient/year	*3968.71*	

*Notes: ∗* for the 2014 fiscal year, the average annual currency rate published by Romanian National Bank was 1 € = 4.4446 lei [22].

## Data Availability

The data used to support the findings of this study are available from the corresponding author upon request.
